# Phenotypic characteristics of circulating tumor cells and predictive impact for efficacy of chemotherapy in patients with pancreatic cancer: a prospective study

**DOI:** 10.3389/fonc.2023.1206565

**Published:** 2023-09-01

**Authors:** Hee Seung Lee, Eun Hye Jung, Hyejung Shin, Chan Su Park, Soo Been Park, Dawoon E. Jung, Galam Leem, So Jung Kim, Jung Hyun Jo, Moon Jae Chung, Jeong Youp Park, Seungmin Bang, Seung Woo Park, Si Young Song

**Affiliations:** ^1^ Division of Gastroenterology, Department of Internal Medicine, Yonsei University College of Medicine, Seoul, Republic of Korea; ^2^ Institute of Gastroenterology, Yonsei University College of Medicine, Seoul, Republic of Korea; ^3^ Biostatistics Collaboration Unit, Medical Research Center, Yonsei University College of Medicine, Seoul, Republic of Korea

**Keywords:** pancreatic ductal adenocarcinoma, circulating tumor cells, prospective study, biomarker, outcome

## Abstract

**Objective:**

Early chemoresistance and tumor mass progression are associated with poor prognosis in pancreatic ductal adenocarcinoma (PDAC). Circulating tumor cells (CTCs) have been studied as potential predictors of treatment response and prognosis in PDAC; however, this approach has yet to be applied in clinical practice. The aim of our study was to investigate the phenotypic characteristics of CTCs and determine their predictive value for PDAC progression.

**Methods:**

We prospectively enrolled 40 patients who were pathologically diagnosed with PDAC and collected blood samples at diagnosis, 2 months after diagnosis, and during disease progression or recurrence. We used a microfabricated filter-based enrichment system to retrieve and analyze CTCs, which were classified using immunofluorescence staining (CD45, EpCAM, and vimentin).

**Results:**

Our study included 20 women and 20 men (median age, 66 years). Overall, 45% of the patients (18/40) had disseminated disease, and 77.5% (31/40) received chemotherapy. Multivariate analysis revealed that the total CTC count and carbohydrate antigen 19-9 level at 2 months after diagnosis were associated with disease progression (*P*<0.05). Linear mixed model analysis revealed that the total CTC count and vimentin-positive CTCs were significantly correlated with treatment response during chemotherapy (*P*=0.024 and 0.017, respectively). Kaplan–Meier analysis showed that total CTC positivity at 2 months was significantly associated with poor progression-free survival (*P*=0.038).

**Conclusion:**

Our study’s findings suggest that CTCs can serve as predictive biomarkers of clinical outcomes in patients with PDAC receiving palliative chemotherapy. In particular, the total CTC count and vimentin-positive CTCs showed changes associated with the chemotherapy response.

## Introduction

Pancreatic ductal adenocarcinoma (PDAC) is a well-known solid tumor with a poor prognosis. Despite technical advancements in early diagnosis, treatment, and cancer management, the 5-year survival rate remains low (1–3). Typically, patients with unresectable PDAC receive chemotherapy, such as FOLFIRINOX (fluorouracil, leucovorin, irinotecan, and oxaliplatin) or gemcitabine/nab-paclitaxel, and the tumor response is evaluated using imaging modalities or tumor markers, such as carbohydrate antigen 19-9 (CA 19-9) and carcinoembryonic antigen (4–6). These blood tumor markers are commonly used to diagnose and monitor the treatment response of patients with PDAC; however, recent studies have shown that circulating tumor cells (CTCs) may offer more valuable insights (7, 8).

CTCs are an uncommon subpopulation of tumor cells found in the peripheral blood of patients with cancer and are induced by tumor angiogenesis. Therefore, CTCs are expected to be involved in tumor invasion and metastasis (9, 10). Previous studies have shown that CTC levels can change during several weeks of treatment and can be used to investigate the effectiveness or resistance of treatments (7, 11, 12). Javed et al. Reported that persistent circulating tumor cells after oncologic resection predict early recurrence in PDAC, with a median time to recurrence of 3.9 months compared to 27.1 months in those without such cells (13). Okubo et al. studied the number of CTCs in patients with advanced PDAC before and after treatment (7). They found that patients with progressive disease had a significantly higher rate of positive CTCs (45.4%) compared to those with stable disease or partial response (24.1%). This suggests that changes in CTCs are linked to the tumor’s response to treatment.

However, prospectively designed sequential blood sampling studies are rare, and the clinical role of CTCs subtypes in PDAC has not yet been established. Therefore, in this study, we aimed to investigate the phenotypic characteristics of CTCs and verify the relationship between the chemotherapy response of PDAC and CTCs by analyzing the count and change in CTC levels, sequentially.

## Materials and methods

### Ethics statement

This study was conducted in accordance with the ethical guidelines of the 1975 Declaration of Helsinki, and all patients provided written informed consent for the use of their blood. This study was also approved by the Institutional Review Board of Yonsei University (IRB approval number: 4-2017-1161) and registered with ClinicalTrials.gov (Identifier: NCT05745415).

### Study design and patients

We prospectively investigated the predictive function of CTCs in patients with PDAC. The study’s inclusion criteria are as follows: (1) Men and women over 20 years of age with a histological diagnosis of PDAC. (2) Eastern Cooperative Oncology Group (ECOG) performance status of 0-1. (3) Life expectancy of greater than 90 days, as assessed by the investigator. (4) Ability to provide informed consent. (5) Presence of measurable disease according to RECIST 1.1 criteria. The exclusion criteria are as follows: (1) Patients who have received prior systemic anti-cancer therapy or are currently receiving anti-cancer therapy. (2) Patients considered unsafe for inclusion in the study by the investigator for any medical or non-medical reason.

### Data collection and definitions

Information on age, sex, tumor location, tumor size, tumor stage, treatment, tumor response, and CA 19-9 levels was collected from electronic medical records. Tumor response was assessed using computed tomography every 2 months. The RECIST (Response Evaluation Criteria in Solid Tumors) criteria define three tumor response categories: Partial Response (PR) indicates a significant decrease in tumor size (typically ≥30%), with no new lesions or tumor progression elsewhere. Stable Disease (SD) refers to no significant change in tumor size, and any changes are within a limited range. Disease Progression (PD) is characterized by a significant increase in tumor size (typically ≥20%), appearance of new lesions, or worsening of existing lesions. Patients with PDAC were divided into two groups based on their response to chemotherapy. Responders were defined as patients whose best response was partial response (PR) or stable disease (SD) after chemotherapy. Non-responders were defined as patients whose best response was progressive disease (PD) after chemotherapy.

Overall survival (OS) was defined as the interval from the date of diagnosis to the date of death due to any cause or the last follow-up. Progression-free survival (PFS) was defined as the time interval from the date of diagnosis to the date of disease progression or death.

### CTC acquisition and detection

**Figure 1:**
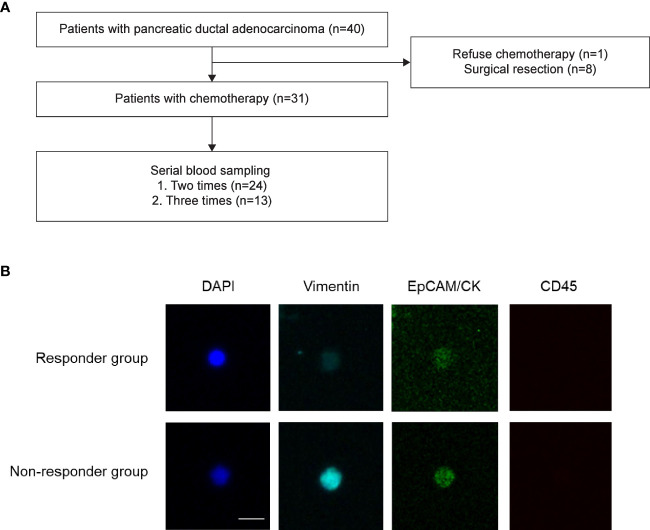
Study flow. **(A)** Patient enrollment flow. Forty patients with pancreatic ductal adenocarcinoma were prospectively enrolled in the present study. **(B)** Representative images of circulating tumor cells. Responder Responders were defined as patients whose best response was partial response or stable disease after chemotherapy. Non-responders were defined as patients whose best response was progressive disease after chemotherapy.

Patient’s blood samples were obtained at the initial diagnosis before treatment to standardize the time of sampling. For sequential blood sampling, we collected samples immediately prior to the next chemotherapy session to reduce the effect of previous chemotherapy on CTCs. Samples were collected three times: at the initial diagnosis (V1), 2 months later (V2), and at the time of tumor progression based on the RECIST criteria (V3) (**Figures 1A, B**). A total of 7.5 mL of blood was obtained and analyzed according to a previous protocol (14). CTCs were isolated using the SMART BIOPSY SYSTEM Isolation kit (cat no. CIKW10; Cytogen, Inc., Seoul, Korea) (15). Briefly, blood samples were incubated with 20 µg/µL of a specifically developed antibody cocktail from the kit, which targets white blood cells (CD45) and red blood cells (globin). Then, the samples were mixed with a pre-activation buffer, followed by density gradient centrifugation at 400 g for 30 minutes at room temperature. Cell suspensions containing CTCs were collected and gradually diluted with a dilution buffer (Cytogen, Inc.). Next, the diluted cell suspensions were filtered through an HDM chip (Cytogen, Inc.), as previously described (**Supplementary Figure 1A**) (14). Cells on the HDM chip were collected and transferred to microtubes.

For immunofluorescence staining, isolated cells were fixed on slides in 4% paraformaldehyde for 5 minutes at room temperature and then stored at 4°C until further processing. Cells on the slides were permeabilized with 0.2% Triton X-100 in phosphate-buffered saline (PBS) for 10 minutes at room temperature. Then, they were blocked with 1% bovine serum albumin in PBS for 60 minutes and incubated with primary antibodies for 60 minutes, followed by incubation with secondary antibodies under the same conditions. The following primary antibodies were used: mouse anti-EpCAM (Cell Signaling Technology, Danvers, MA), mouse anti-cytokeratin (Sigma-Aldrich, St. Louis, MO), rabbit anti-vimentin (Thermo Fisher Scientific, Waltham, MA), and rabbit anti-CD45 (Cell Signaling Technology). The secondary antibodies used were goat anti-rabbit Alexa Fluor 647 (Thermo Fisher Scientific) and goat anti-mouse Alexa Fluor 488 (Thermo Fisher Scientific). The slides were mounted using the Fluoroshield Mounting Medium with DAPI (ImmunoBioScience Corp.), stained cells were observed, and images were captured using a fluorescence microscope (Eclipse Ti; Nikon Corporation, Tokyo, Japan) with a 400× objective. We defined the total CTC-positive cells as more than 2 CTCs in the patient’s blood.

### Statistical analyses

Descriptive statistics are presented as medians (first quartile, third quartile) or numbers (percentages). The Mann–Whitney U test was performed to test group differences in continuous variables. Various types of continuous CTC variables and the CA19-9 level were measured twice. These outcomes were analyzed using the linear mixed model method to assess the interaction effect between group and time. A compound symmetry covariance structure was assumed to address the within-subject effect. The association between survival outcomes and the explanatory variables of the CTC variable at each time point and baseline characteristic variables was evaluated using Cox regression analysis. Variables with *P*-values <0.05 in the univariate analysis were examined by multivariate analysis. The survival rate was estimated using the Kaplan–Meier method and compared between groups using the log-rank test.

Statistical analyses were conducted using SAS, version 9.4 (SAS Institute, Cary, NC) and R package software (version 4.0.4, http://www.r-project.org/). Statistical significance was set at *P*<0.05.

## Results

### Patient characteristics

We analyzed 78 blood samples from 40 patients (**Figure 1**). Baseline characteristics of the patients are presented in **Table 1**. Our study included 20 women and 20 men (median age, 66 years). Among the 40 patients, 9 (22.5%) were resectable and underwent surgical treatment. Overall, 45% of the patients (18/40) had disseminated disease, and 77.5% (31/40) received chemotherapy as the initial treatment. Among the 13 patients who were treated with the gem-based regimen, 12 patients received gemcitabine plus nab-paclitaxel, and 1 patient received gemcitabine plus erlotinib.

**Table 1:** Patients’ demographics.

Variable	N=40
Age, y	66 (61, 75)
Male sex	20 (50.0%)
Location	
Head	22
Body	10
Tail	8
Tumor size, mm	33
Stage	
I	9 (22.5%)
II	7 (17.5%)
III	6 (15.0%)
IV	18 (45.0%)
Chemotherapy	
FOLFIRINOX	18
Gemcitabine-based chemotherapy	13
Tumor response after 2 months of chemotherapy	
Progression	6 (19.4%)
Stable disease	15 (48.4%)
Partial response	5 (16.1%)
CA 19-9 level, IU	426 (68.3, 2894)

Variables are expressed as n (%) and median (first quartile, third quartile).

Resectability was assessed according to the National Comprehensive Cancer Network guideline.

FOLFIRINOX (fluorouracil, leucovorin, irinotecan, and oxaliplatin)

CA 19-9, carbohydrate antigen 19-9.

### Disease progression-related risk factors during chemotherapy

Among 31 patients who received chemotherapy, 19 (61.3%) showed disease progression. Risk factors for disease progression were analyzed using univariate and multivariate Cox regression analyses of data obtained from patients undergoing chemotherapy. Univariate analysis indicated statistical significance in metastasis at diagnosis, V2 CTCs, and V2 CA 19-9 (all, *P*<0.05). Multivariate analysis indicated the statistical significance of V2 CA 19-9 (*P*=0.047) and near statistical significance of metastasis at diagnosis (*P*=0.066) and V2 CTCs (*P*=0.056) (**Table 2**).

**Table 2:** Results of univariate and multivariate analyses according to disease progression-related risk factors in patients who underwent chemotherapy.

	Univariate analysis			Multivariate analysis	
	HR (95% CI)	*P*-value	C-index (95% CI)	HR (95% CI)	*P*-value
Age	1.007 (0.959, 1.058)	0.774	0.518 (0.348, 0.688)		
Women vs. men	1.182 (0.475, 2.942)	0.719	0.525 (0.400, 0.650)		
Metastasis at diagnosis	3.751 (1.258, 11.184)	0.017	0.627 (0.509, 0.746)	3.440 (0.920, 12.867)	0.066
Distant node (+)	2.352 (0.760, 7.278)	0.138	0.561 (0.466, 0.656)		
V1 total CTCs, yes vs. no	0.889 (0.325, 2.434)	0.819	0.502 (0.373, 0.631)		
V1 tumor size, mm	1.007 (0.976, 1.039)	0.668	0.529 (0.387, 0.671)		
V1 CA 19-9 level, U/mL	1.004 (0.998, 1.011)	0.219	0.604 (0.455, 0.753)		
V2 total CTCs, yes vs. no	3.122 (1.009, 9.658)	0.048	0.658 (0.556, 0.761)	3.250 (0.970, 10.896)	0.056
V2 tumor size, mm	1.018 (0.991, 1.045)	0.198	0.628 (0.482, 0.774)		
V2 CA 19-9 level, U/mL	1.016 (1.006, 1.025)	0.001	0.683 (0.533, 0.833)	1.012 (1.000, 1.025)	0.047
Gemcitabine-based chemotherapy vs. FFX	1.552 (0.591, 4.073)	0.372	0.545 (0.413, 0.677)		

HR, hazard ratio; CI, confidence interval; vs., versus; CTCs, circulating tumor cells; CA 19-9, carbohydrate antigen 19-9; V1, at the initial diagnosis; V2, 2 months later; FFX, FOLFIRINOX.

### Correlation between the number of CTCs and treatment response

**Figure 2:**
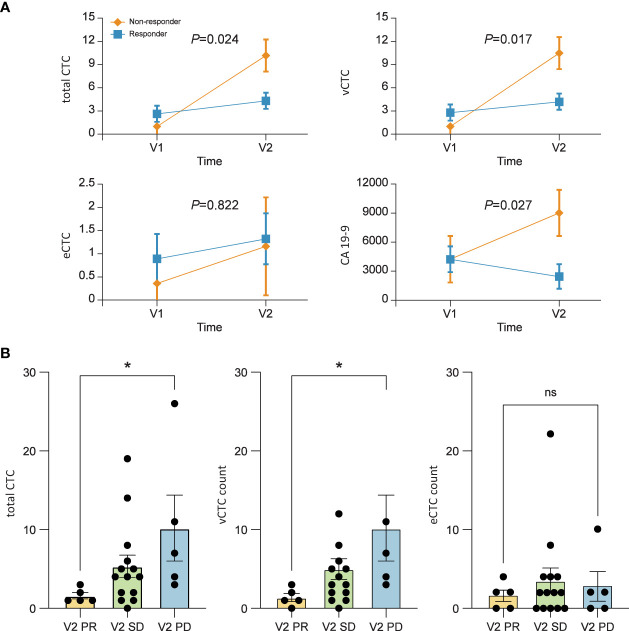
**(A)** Mean profile plot. Linear mixed model to find the correlation between treatment response and circulating tumor cell (CTC) counts. The changing pattern of CTCs over time according to the tumor response. The levels of total CTCs (*P*=0.024), vimentin-positive CTCs (vCTCs) (*P*=0.017), and carbohydrate antigen 19-9 (*P*=0.027) are significantly correlated with patient prognosis. **(B)** The numbers of total CTCs and vCTCs after 2 months of chemotherapy are significantly higher in patients who showed tumor progression than in those with a partial response. Values represent the estimated means with standard error from linear mixed models.

The changing patterns of total CTCs and vimentin-positive CTCs (vCTCs) over time revealed a difference between the responder and non-responder groups. As shown in **Figure 2A**, the slopes of the lines were significantly higher in the non-responder group than in the responder group (total CTCs, *P*=0.024; vCTCs, *P*=0.017). The tumor marker CA 19-9 level also increased in the non-responder group and decreased in the responder group from visit 1 to visit 2 (*P*=0.027). However, the EpCAM-positive CTC (eCTC) did not show an association between change in the number of CTCs and treatment response (*P*=0.822). We also compared the number of CTCs between the V2 partial response group and disease progression group. The total number of CTCs and vCTCs was significantly higher in the disease progression group than in the partial response group (median number 7 vs. 1, *P*=0.015) (**Figure 2B**).

### Overall survival and progression-free survival according to CTC positivity

**Figure 3:**
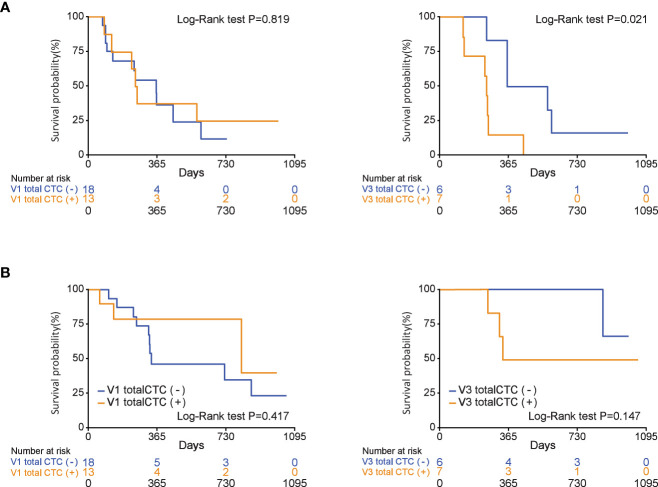
During chemotherapy, total circulating tumor cell (CTC) positivity is associated with progression-free survival in patients who were diagnosed with pancreatic ductal adenocarcinoma (V3 total CTCs (+), P=0.021). **(A)** Progression-free survival, **(B)** overall survival.

The OS and PFS according to the total CTC status at three different time points (V1, V2, and V3) are shown in **Figure 3** and **Supplementary Figure 2**. PFS was significantly longer in patients with a negative total CTC after 2 months of chemotherapy than in those with positive total CTCs (median PFS, 447 vs. 240 days, *P*=0.038). Regarding OS, V2 total CTC positivity did not show a survival difference (median OS, not reached vs. 336 days, *P*=0.065). One-year survival rates of patients with negative and positive total CTCs were 86% and 42%, respectively.

### Association between the total CTC count and clinical tumor status

**Figure 4:**
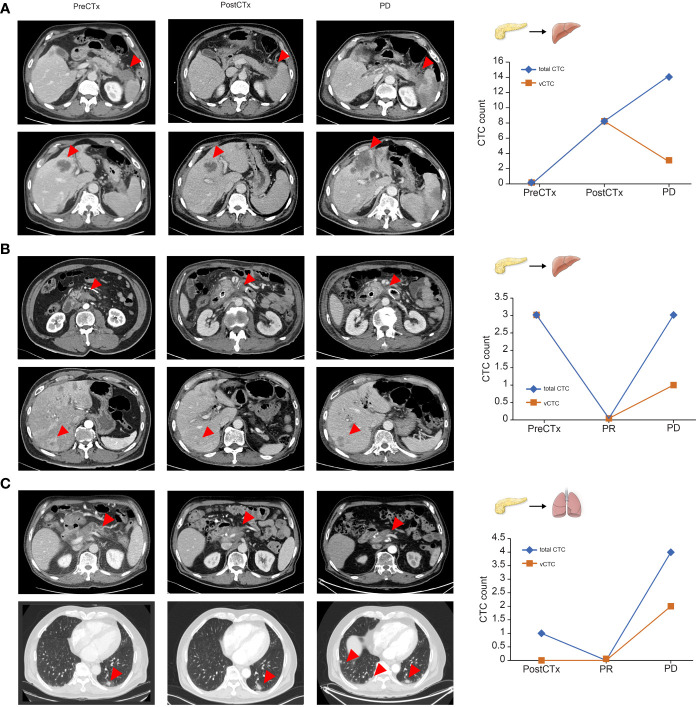
Representative cases of circulating tumor cell dynamics and correlation with treatment response **(A–C)**. Arrows represent primary pancreatic tumors and metastatic lesions.

As shown in the representative patient example in **Figures 4A–C**, we analyzed the trend of changes in the number of CTCs in patients before chemotherapy, 2 months after chemotherapy, and after disease progression. In representative cases, the total number of CTCs reflected the treatment response in patients with PDAC receiving chemotherapy.

## Discussion

Our study was designed to investigate the association between the number of CTCs in the blood of patients with pancreatic cancer and their response to chemotherapy and prognosis. Blood samples were collected from 40 patients at three time points: diagnosis, 2 months after diagnosis and treatment, and at the time of progression. Therefore, it was confirmed that there was a significant difference in the total number of CTCs between those who progressed 2 months after diagnosis and those who did not. Additionally, when the three time points were viewed serially, the tendency of CA 19-9 and CTC values to increase over time differed between the progression group and non-progression groups. The presence of CTC was associated with PFS, but not with OS. Subsequent chemotherapies may significantly influence the final survival duration in pancreatic cancer. Additionally, it is essential to consider that the prognosis of pancreatic cancer is fatal, and unlike PFS numerous factors can contribute to patient outcomes, such as tumor size, age, sex, and tumor characteristics. Therefore, it is challenging to attribute the patients’ survival solely to the presence of circulating tumor cells. In previous study, median OS was significantly correlated with the percentage of patients who received subsequent chemotherapy after first line chemotherapy of their disease (16). Another study also reported that the positivity of CTCs was not predictive of decreased OS but was associated with tumor recurrence (17).

Considering the fatal prognosis of pancreatic cancer due to unnoticeable early metastasis, CTCs are considered good candidates for detecting distant invasion and metastasis (18). According to a meta-analysis, CTC-positive patients had shorter OS and PFS than CTC-negative patients (19). Pilot studies have demonstrated that CTCs can also be detected in the premalignant stages of pancreatic cancer; however, larger confirmatory studies are required (20). These data suggest that CTCs may serve as predictive biomarkers for pancreatic cancer before treatment (19). However, the relationship between the prognosis of pancreatic cancer and CTCs remains controversial, with inconsistent results in other studies. Recent studies have also focused on the heterogeneity of CTCs to classify and analyze their characteristics (21–23). For the last three decades, technological advancements in the detection and characterization of CTCs have allowed us to understand their function in metastatic processes (24). To characterize cell populations, mesenchymal markers, such as vimentin and N-cadherin, and markers related to stemness, such as CD133, CD44, and ALDH, have been used (25). Our study also analyzed CTCs by dividing not only total CTCs, but also vimentin-positive CTCs and EpCAM-positive CTCs. Additionally, reliable results were obtained by analyzing CA19-9, which is widely used for prognostic analyses in clinical practice.

Cancer cells often lose their epithelial characteristics and acquire features of a more mesenchymal phenotype, a process referred to as epithelial-to-mesenchymal transition (EMT) (26). Some CTCs undergo EMT, resulting in the down-regulation of cytokeratin expression (27). In our study, the significant association of vimentin-positive CTCs with prognosis highlights the potential importance of mesenchymal-type CTCs in pancreatic cancer management. Mesenchymal-type CTCs are known to possess unique characteristics that may contribute to cancer progression, metastasis, and treatment resistance (25). Understanding the biological features of these CTCs can provide valuable insights into the disease’s aggressive behavior and guide treatment decisions, particularly given the highly active EMT process in PDAC. The presence and significance of vimentin-type CTCs in the bloodstream hold particular importance in this context. However, it is also important to acknowledge the limitations associated with the detection and analysis of mesenchymal-type CTCs. Their relative rarity and dynamic nature in circulation can present challenges in their reliable identification and quantification. Additionally, the clinical implications of mesenchymal-type CTCs should be interpreted in the context of other prognostic factors and tumor characteristics.

In addition to CTCs, circulating tumor DNA (ctDNA) has been widely investigated in the prognostic studies of pancreatic cancer. According to a recent systematic review, increased detection of ctDNA showed a tendency toward more aggressive tumor behavior and decreased OS and PFS (28). However, they have not yet entered clinical routine owing to their inaccessibility and lack of standardized methods for detection. Among the metastatic subgroups, no correlation was found between the number of *KRAS* mutations and PFS or OS (29). Despite the high prevalence of *KRAS* mutations in pancreatic cancer, the ctDNA detection rates are 50–75% in metastatic patients with pancreatic cancer, which is lower than that in patients with breast or lung cancers, whose detection rate is usually less than 70–80% (30). The use of ctDNA in advanced-stage disease is also less appealing because of the low rate of targetable mutations associated with Food and Drug Administration (FDA)-approved medications (31).

Contrary to expectations, there was no significant difference between the stages, and the number of CTCs did not show a tendency to be higher in chemotherapeutic patients than in surgical patients. This may be problematic because of the small sample size and enrollment of patients at only one institution. As the characteristics of cancer differ among patients, it is difficult to simply compare the numbers. Comparisons within same patients may be more important than CTCs number comparisons between patients. Even if the difference between patients is small, it is meaningful because the number of CTCs tends to increase in cases of metastasis, recurrence, or progression within a patient. CTC identification rates of approximately 5–40% in several studies using the FDA-approved CellSearch® technology were fairly underwhelming for patients with PDAC (32–34). In our study, the detection rate of CTCs was not very high; therefore, there was a limitation in the statistical analysis. The cut-off number of CTCs was different for each study, and there is no unified standard yet. Usually, the cut-off number of CTCs dividing CTC positivity/negativity is ≥3 CTCs/4 mL (12, 35) or ≥2 CTCs/4 mL (36). In our study, a cut-off of ≥3 CTCs/7.5 mL had meaningful results when comparing the progression and non-progression groups. And, validation through flow cytometry is necessary to confirm the presence of circulating tumor cells. Large-scale prospective studies are needed to clarify the issue of CTCs counts and its impact on chemo-response prediction in the future.

Despite these limitations, our study obtained significant results by comparing and analyzing the number of CTCs in patients with pancreatic cancer by dividing the patients into those with and without progression at a single time point. Moreover, it is meaningful in that it was possible to see the change in the CTC count of each patient by serially tracking 40 patients and collecting the CTC count at different time points. Through this, a comparison of the number of CTCs within each patient was meaningful. In our study, not only the number of CTCs but also the CA19-9 levels of the patients were measured, confirming a similar trend. Similar to other studies, this approach has the advantage of obtaining more accurate results when predicting the prognosis of patients with pancreatic cancer by combining the CA19-9 level and CTCs (19, 21).

In conclusion, we confirmed the significant correlation between the number of CTCs and advanced pancreatic cancer. In addition, it was confirmed that the ratio of the number of CTCs increased over time in the group with poor prognosis compared to the group with a good response to treatment. Thus, it is expected that CTCs can help predict the prognosis of patients with pancreatic cancer.

## Data availability statement

The data generated in this study are available upon request from the corresponding author.

## Ethics statement

The studies involving humans were approved by Institutional Review Board of Yonsei University (IRB approval number: 4-2017-1161). The studies were conducted in accordance with the local legislation and institutional requirements. The participants provided their written informed consent to participate in this study.

## Author contributions

HL, SS: study conception and design; EJ, HS, GL, SK, JJ, MC, JP, SB, SWP, SS, HL: data acquisition, and data analysis and interpretation; EJ, HS, HL: manuscript writing and data analysis; All authors: final review; GL, SK, JJ, MC, JP, SB, SWP, SS, HL: patients enrollment and data review; EJ, HS: Statistical analysis; SBP, DJ: Sample preparation and Experimental analysis.
