# Supplementary material for: Phenotypic characteristics of circulating tumor cells and predictive impact for efficacy of chemotherapy in patients with pancreatic cancer: a prospective study
Source: Front Oncol. 2023 Sep 1;13:1206565. doi: 10.3389/fonc.2023.1206565 (PMC10509470; doi:10.3389/fonc.2023.1206565)
Supplement: Supplementary file 1 [file DataSheet_1.pdf]

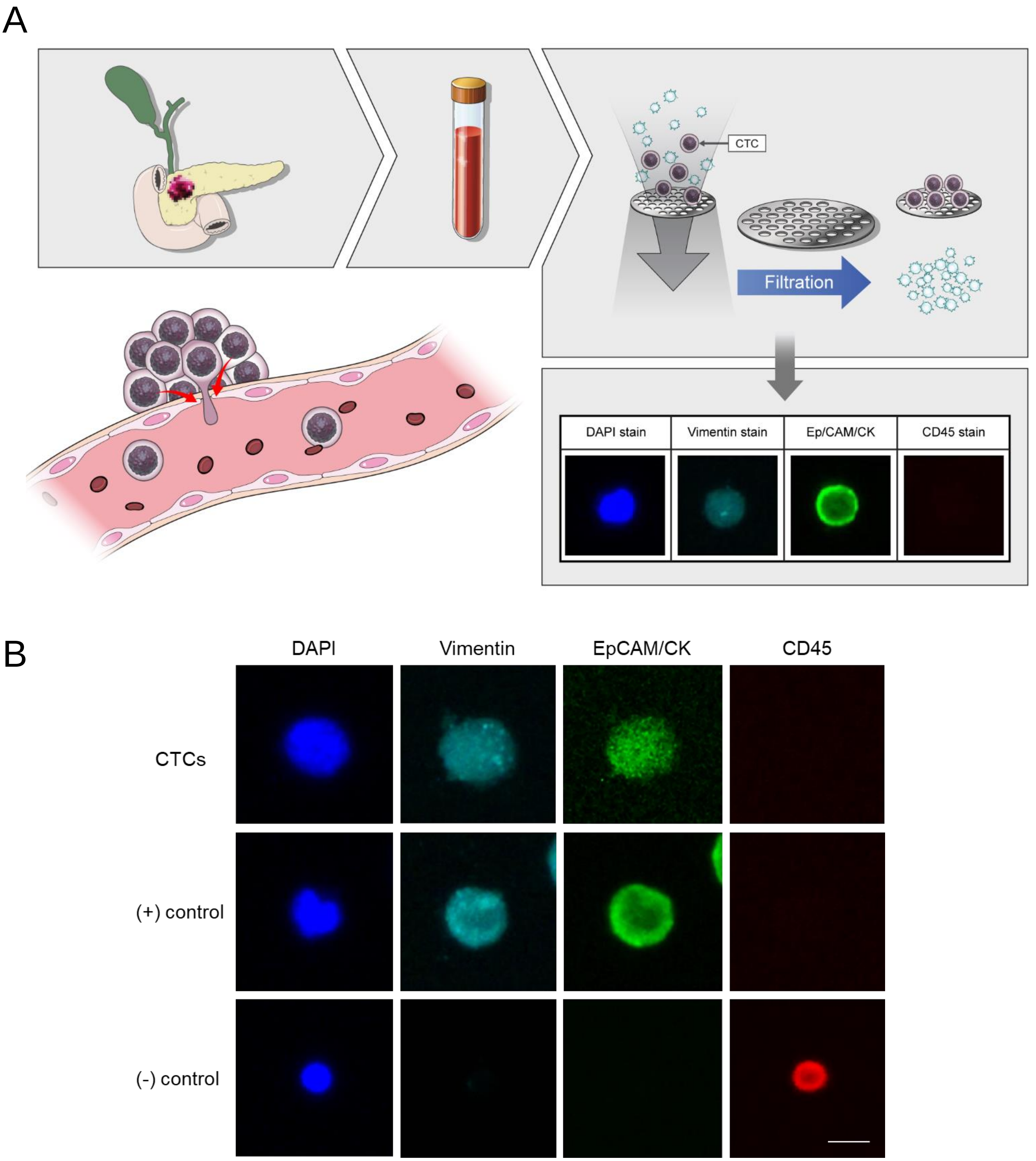

Supplementary Figure 1. (A) Circulating tumor cell (CTC) separation and detection process. After serial blood sampling, CTCs were filtered through an HDM chip (Cytogen, Inc., Seoul, Korea). Epithelial and mesenchymal type CTCs were separated and counted to investigate the association with their predictive impact on the efficacy of chemotherapy. (B) Representative image of circulating tumor cells. The positive control is CFPAC-1 cell lines (EpCAM, vimentin positive), and negative control was PBMC (CD45 positive). Scale bar: 10 µm.
