# Supplementary material for: Phenotypic characteristics of circulating tumor cells and predictive impact for efficacy of chemotherapy in patients with pancreatic cancer: a prospective study
Source: Front Oncol. 2023 Sep 1;13:1206565. doi: 10.3389/fonc.2023.1206565 (PMC10509470; doi:10.3389/fonc.2023.1206565)
Supplement: Supplementary file 2 [file DataSheet_2.pdf]

A

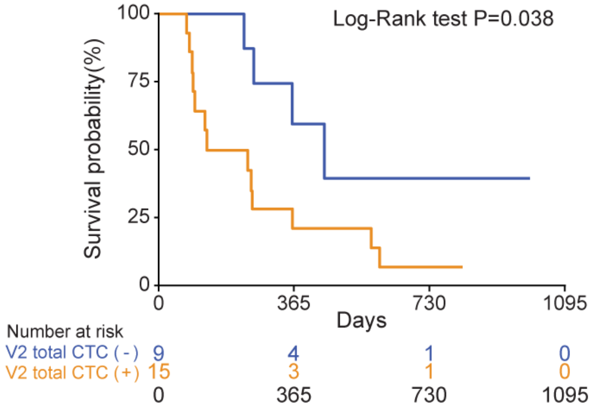

B

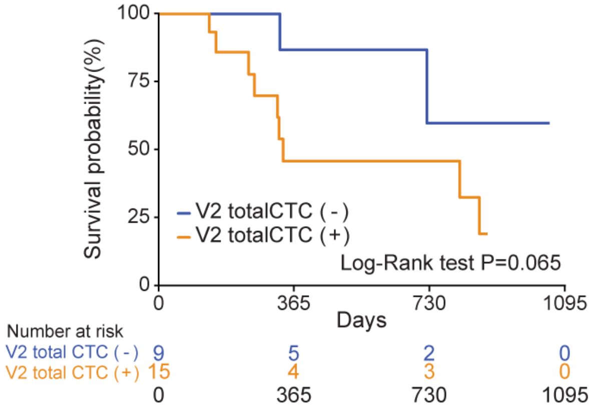

Supplementary Figure 2. During chemotherapy, total circulating tumor cell (CTC) positivity is associated with progression-free survival in patients who were diagnosed with pancreatic ductal adenocarcinoma (V2 total CTCs (+), P=0.038). (A) Progression-free survival, (B) overall survival.
